# Development of a Multiplexing Injector for Gas Chromatography for the Time-Resolved Analysis of Volatile Emissions from Lithium-Ion Batteries

**DOI:** 10.3390/molecules29102181

**Published:** 2024-05-07

**Authors:** Maria Antoniadou, Valentin Schierer, Daniela Fontana, Jürgen Kahr, Erwin Rosenberg

**Affiliations:** 1 Institute of Chemical Technologies and Analytics, Vienna University of Technology, Getreidemarkt 9/164, A-1060 Vienna, Austria; 2 Electric Drive Technologies, Electromobility Department, Austrian Institute of Technology GmbH, Giefinggasse 2, A-1210 Vienna, Austria; valentin.schierer@tuwien.ac.at (V.S.); juergen.kahr@ait.ac.at (J.K.); 3 FAAM Research Centre, Strada del Portone 61, I-10137 Torino, Italy

**Keywords:** gas chromatography, multiplexing, multiplex sampling, time-resolved measurements, lithium-ion batteries

## Abstract

Multiplex sampling, so far mainly used as a tool for S/N ratio improvement in spectroscopic applications and separation techniques, has been investigated here for its potential suitability for time-resolved monitoring where chromatograms of transient signals are recorded at intervals much shorter than the chromatographic runtime. Different designs of multiplex sample introduction were developed and utilized to analyze lithium-ion battery degradation products under normal or abuse conditions to achieve fast and efficient sample introduction. After comprehensive optimization, measurements were performed on two different GC systems, with either barrier discharge ionization detection (BID) or mass spectrometric detection (MS). Three different injector designs were examined, and modifications in the pertinent hardware components and operational conditions used. The shortest achievable sample introduction time was 50 ms with an interval of 6 s. Relative standard deviations were lower than 4% and 10% for the intra- and inter-day repeatability, respectively. The sample introduction system and column head pressure had to be carefully controlled, as this parameter most critically affects the amount of sample introduced and, thus, detector response. The newly developed sample introduction system was successfully used to monitor volatile degradation products of lithium-ion batteries and demonstrated concentration changes over the course of time of the degradation products (e.g., fluoroethane, acetaldehyde and ethane), as well as for solvents from the battery electrolyte like ethyl carbonate.

## 1. Introduction

Gas chromatography is the primary technique for the analysis of volatile and semi-volatile organic compounds in a large variety of environmental, industrial, biological or food/flavor-related samples. Its versatility is a consequence of the numerous options this technique offers for sample introduction, separation and detection that allow the customization of a GC system to the particular requirements [1]. Depending on whether qualitative or quantitative analysis is the aim, specific detectors such as mass spectrometry (MS) or universal detectors such as flame ionization (FID), thermal conductivity (TCD) or barrier discharge-ionization detection (BID) can be used. This latter detector, being a relatively recent addition to the range of gas chromatographic detectors [2], is an attractive alternative to both the FID and the TCD in that it offers sensitive, non-destructive and universal detection even of analytes that do not or hardly respond in flame ionization detection. It has thus been used in a variety of applications [3,4,5,6,7,8,9], including the analysis of volatile degradation products of the organic electrolyte of lithium-ion batteries [10,11].

Separation techniques are typically used where several analytes have to be determined simultaneously in complex matrices or where the interference-free determination of one or more analytes requires their separation from the matrix. Since separation is achieved in time (rather than in space), chromatography is a technique that—in its classical format—cannot be used for continuous monitoring of process streams. Various strategies have therefore been developed to overcome this shortcoming. Among the more common approaches are the use of short columns or miniaturized separation systems [12], separations under vacuum outlet (low pressure) conditions [13], low thermal mass/directly resistively heated systems and very recently, also negative thermal gradient GC systems [14].

A totally different approach that provides high(er) temporal resolution for chromatographic analyses is the use of multiplexed or multiplexing chromatography [15]. The core of this technique is that a sample is introduced into the separation system at predefined intervals. The samples are separated, and as the duration of a single separation is longer than the interval between sample injections, the individual chromatograms overlap, leading to a complex signal as a response. This signal must then be deconvoluted to arrive again at the individual chromatograms or an average chromatogram (Supplementary Materials, Figure S1).

**Figure 1:**
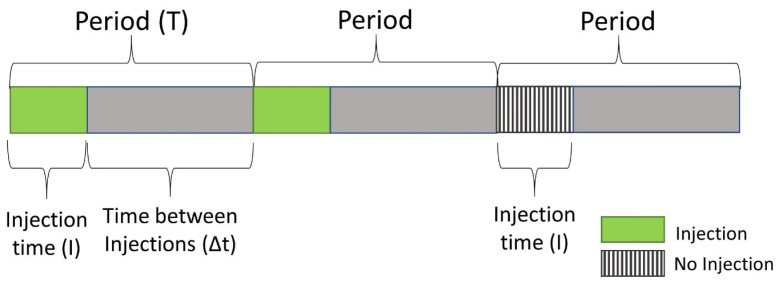
Schematic illustration of the terminology related to modulated injection sequences.

The conditions that are to be fulfilled for successful multiplexing chromatography are the following:

(a) The sample is introduced at irregular intervals into the separation system according to a pre-defined so-called ‘pseudo-random binary sequence’ (PRBS) consisting of only “0” and “1” values where the former codes no sample introduction, while the latter stands for the introduction of sample;
(b) The interval of sample introduction (I) is much shorter than the interval between injections, the period T, and also the width of each individual signal (Figure 1);
(c) Chromatographic conditions must be stationary so that each injected sample is exposed to exactly the same separation conditions. This means in practice that separations have to be performed in the isocratic (for HPLC) or in the isothermal mode (for GC), which limits its practical applicability to samples with a relatively narrow polarity range (HPLC) or boiling point distribution (GC).

When these conditions are fulfilled, and additionally, the number of data points that have been acquired can be expressed as 2*^n^* (ranging from 0…*m* = 2*^n^*−1), then it is possible to deconvolute the data using the Hadamard transform, or rather, the corresponding back-transformation. The Hadamard transformation is, similar to the Fourier transformation, an ideally loss-free linear transformation from one into another data space. It is typically used for data compression, signal processing and error correction [16]. It explains the formation of the convoluted chromatogram (represented by a vector of dimensionality *m*) by the product of the so-called convolution matrix [***S***] (a *m* × *m* matrix derived from the PRBS (Equation (1)) [17]

(1) $$\left[S\right]\times \left[\begin{array}{c} deconvoluted\\ chromatogram \end{array}\right]=\left[\begin{array}{c} convoluted\\ chromatogram \end{array}\right]$$

The deconvoluted chromatogram was obtained by multiplying the inverse convolution matrix with the convoluted chromatogram, as shown in Equation (2) (inverse Hadamard transformation):

(2) $${\left[S\right]}^{-1}\times \left[\begin{array}{c} convoluted\\ chromatogram \end{array}\right]=\left[\begin{array}{c} deconvoluted\\ chromatogram \end{array}\right]$$

The most important motivation for using multiplexing chromatography is to improve the signal-to-noise ratio of weak signals. While the signal increases *n* times with an *n*-fold performance of a measurement, the noise increases only by a factor of $\sqrt{n}$, therefore also improving the signal-to-noise ratio by a factor of $\sqrt{n}$ (known as ‘Fellgett advantage’, particularly in spectroscopy).

Generally, the application of multiplexing chromatography to improve time resolution rather than the signal-to-noise ratio is less considered but equally useful. In that case, injections are made from a sample stream whose concentration changes during the measurement sequence, and the objective is to represent these temporal changes with a better time resolution than the cycle time, which, particularly in temperature-programmed operation, can be considerably longer than the chromatographic run time [18]. Depending on the complexity of the resulting chromatogram, and particularly the degree of overlap of the individual signals, different strategies can be applied to deconvolute the data and to derive individual chromatograms in which not only the number of peaks and their retention time is correctly determined, but also their intensity or peak area. In case of a strong overlap, deconvolution of the convoluted chromatogram is required according to the inverse Hadamard transformation outlined above. In that case, the condition of non-periodicity of the sample introduction must be fulfilled, as otherwise, it is not possible to deconvolute the data. Data evaluation is based on the calculation of the average chromatogram as an intermediate result [19]. This chromatogram is then used to calculate the concentration profiles of the individual analytes over time. In the case of only partly overlapping or non-overlapping signals resulting from multiplex injection, there is no need for an inverse Hadamard transformation for the deconvolution of the data, and simpler algorithms can be applied. In the simplest case, the peaks do not overlap and can be evaluated directly if their position is known. To this end, it is no longer a requirement that the sample is introduced in a non-periodic sequence (e.g., the previously discussed PRBS), but on the contrary, it largely simplifies data processing if the sample is introduced in periodic intervals, which makes it easier to determine the relevant peak position.

The algorithm proposed in this work is more universally applicable: It relies on fitting the peak profiles of the individual peaks to the convoluted chromatogram. Provided the chromatographic system is not overloaded, the peak width remains essentially constant with the peak height scales and the concentration. This scaling factor is determined in the process of peak fitting, and the resulting peak is subtracted, while the scaling factor is a measure of its relative concentration. The positions at which the scaled peak is subtracted from the complex chromatogram are given by the retention time of the particular analyte in the first chromatogram under consideration of the time interval between injections (and whether an injection was completed or not in the case of non-periodic sample introduction sequences). This process is repeated for each signal in the initial chromatogram. Evidently, all analytes to be determined must already be present in the first chromatogram (but they may be absent in subsequent chromatograms). The algorithm was shown to work satisfactorily when overlapping peaks had a resolution of at least *R* = 1 and when the peak shape of individual peaks did not change during the multiplexed chromatogram as an effect of overload or column wear (see Figure S2 in the Supplementary Materials) [20].

Multiplexing chromatography requires particular consideration of how the sample introduction is achieved. Ideally, sample introduction is undertaken in a narrow injection pulse with a rectangular peak profile. This can be effected more easily in liquid phase separation (high-performance liquid chromatography, HPLC, and capillary electrophoresis, CE), where the sample is typically introduced through an injection valve with a sample loop of appropriate dimensions (HPLC [21,22]) or through either hydrodynamic or electrokinetic injection in CE [23]. In gas chromatography, the situation is more demanding: In the case of liquid sample introduction, ultrafast introduction and evaporation of the sample must be achieved, which calls for dedicated injector designs [24]. Although with gaseous samples, the use of a six-port valve with a gas sampling loop also appears an attractive option, there are two obstacles: First, multiplexing chromatography with high time resolution (Δ*t* = 2…3 s) could mean a chromatographic run of a half hour up to 900 switching cycles, which is a huge number of switching cycles in a very short time, and this could lead to premature leakage and failure of the injection valve. And even then, the typical arrangement of the gas sampling valve upstream of the injector is not suitable for this type of operation if no particular precautions are taken. Depending on the instrument type, the volume of a classical split/spitless injector is in the order of 600–950 µL. Due to this relatively large volume, the injector creates the same effect as if a gaseous sample was introduced into a continuous stirred-tank reactor (CSTR). The characteristics of a CSTR are that with a delta input function (=controlled sample introduction during a short interval), the output function (=transfer of the analytes to the analytical GC column) would have a very steep left edge, while the right edge would follow exponential decay. This is unsuitable for multiplexing injection, which requires narrow, well-defined injection pulses; however, this situation is improving (at the cost of sensitivity) when a high split ratio is used. All these aspects are addressed by the proprietary design of an injector for gaseous samples where the actual location of sample introduction is within the injector in the immediate vicinity of the GC column head. Furthermore, the three designs investigated in this study (design A–C) replace the switching of a six-port valve for sample introduction by the opening and closing of solenoid valves (which are certified to be good for several hundred thousand switching cycles) for the controlled introduction of a defined sample volume. In contrast to this, examples reported in the literature mostly still make use of a six-port valve for sample introduction:

Published examples of multiplex-GC analyses include the detection of volatile organic compounds in indoor air [25], ethanol or toluene in exhaled breath after drinking or smoking [26], acetone in human breath [27] and hexamethyldisiloxane in a wafer cleanroom [24]. Recently, the use of switching valves in combination with column switching techniques has been used for fast sample introduction in the monitoring of catalytic reactions [28,29]. In general, a multiplex sample introduction is interesting for all applications where fast reactions take place and where a better time resolution than one data point per GC cycle time is required. One prominent example is the case of lithium-ion battery degradation products.

Lithium-ion batteries (LIBs) are widely used as an energy storage. To better understand the degradation mechanisms of the organic electrolyte and to improve the safety and performance of LIBs, qualitative and quantitative analysis with high-time resolution is needed. Different chromatographic techniques have already been used for the analysis of electrolyte degradation products [30]. The GC methods use temperature programs that span a wide temperature interval and consequently lead to long cycle times, including the cooling phase. Also, direct MS analysis is not a suitable option in this case, as there are important isobaric interferences that would not be resolved (C_2_H_4_/CO and CH_3_CHO/CO_2_, respectively) [31].

The current work addresses the development and investigation of three multiplex injector configurations based on headspace sampling and the introduction of gaseous compounds. Various parameters that can affect the analytical performance are examined and evaluated according to the relative standard deviation (RSD%) and peak area response values. To demonstrate the feasibility of our prototype multiplexing injector, we performed an in situ analysis of lithium-ion battery degradation products and obtained time-resolved chromatograms for a number of relevant compounds.

## 2. Materials and Methods

### 2.1. Materials

All reagents had a purity of at least 95%. *n*-heptane was purchased from Fluka (Buchs, Switzerland). Ethanol was obtained from Merck (Darmstadt, Germany), acetonitrile and dimethyl carbonate (DMC) battery grade from Sigma Aldrich (Vienna, Austria) and ethyl methyl carbonate (EMC) Selectilyte from BASF (Ludwigshafen, Germany). Helium, used as the GC carrier and BID discharge gas, was of purity ≥99.999% and was purchased from Messer (Gumpoldskirchen, Austria). All battery cell components were provided by Lithops S.r.l., now FAAM Research Center (Torino, Italy), and the samples were prepared in a commercially available test cell for the in situ analysis of gas species in Li-ion systems, the ECC-DEMS (EL-CELL, Hamburg, Germany).

### 2.2. Instrumentation

The instrumentation used for the investigation of different multiplex injector configurations was a GC-2010 Plus gas chromatograph with a Tracera BID barrier-discharge ionization detector equipped with an external 6-port switching valve from Shimadzu (Kyoto, Japan). The column was a DB-5MS (5% phenyl-95% methyl-polysiloxane) 30 m × 0.25 mm × 0.25 μm from Agilent J&W. A GC-MS-QP2010 Plus instrument (Shimadzu) equipped with a Rt-Q-BOND (100% divinylbenzene, Restek, Bellefonte, PA, USA) PLOT (porous layer-open tubular) column of 30 m × 0.32 mm × 10 μm dimension equipped with a particle trap and a guard column was used for real sample analysis. For the laboratory-made injector, an Arduino Leonardo ETH board (RS Components, Frankfurt am Main, Germany) with a code written in-house for Arduino Software (IDE) v1.8.9 (https://www.arduino.cc/en/main/software, accessed on 30 December 2023) was used for the control of the solenoid valves. The normally open 2-way, normally closed 2-way and 3-way solenoid valves used for the work were purchased from Bürkert Austria GmbH (Vienna, Austria). Tee connectors were from VICI Valco (Schenkon, Switzerland).

### 2.3. Analytical Procedure

Sample injection was performed with the laboratory-made injectors as described below. The final method used with the BID detector was an injector temperature of 250 °C, linear velocity of 22.7 cm s^−1^, split of 10:1, discharge gas flow of 50 mL min^−1^, column head pressure of 85.6 kPa, total flow of 14.0 mL min^−1^, column flow of 1.00 mL min^−1^, purge flow of 3 mL min^−1^, oven temperature of 35 °C isothermal, and BID temperature of 300 °C. The analysis runtime varied depending on the sequence length from 5 to 100 min as all data were stored in one single data file. The method used on the GC-MS instrument had an injector temperature of 270 °C, split of 8:1, column head pressure of 87 kPa, column flow of 2.22 mL min^−1^, purge flow of 1 mL min^−1^, oven temperature program of 100 °C, ion source temperature of 220 °C, interface temperature of 220 °C, and MS scan range of 30 to 300 *m*/*z*.

### 2.4. Multiplex Injector Configurations

Three designs for a multiplex injector were developed and examined. These were the one-valve (A), the two-valve (B) and the three-valve (C) design. Although some of the injector designs could have equally been realized with a six-port switching valve, preference was given to solenoid on/off- or three-way valves due to the longer durability, keeping in mind that a single chromatographic run could require several hundred switching cycles and that during regular operation, tens of thousands of switching cycles could be performed within few days at which regular six-port valves often fail. The general idea of the first two sample introduction device designs was that the gaseous sample was introduced through a fused silica capillary into the GC’s injector, which itself was confined within or connected to a stainless-steel capillary. The third introduction device consisted of tubing connections and a stainless-steel sample loop. Depending on the pressure conditions in the injector, at the head of the fused silica capillary and the stainless-steel capillary, and, of course, the valve switching, the sample was or was not introduced into the GC injector and transferred to the GC column.

**Figure 2:**
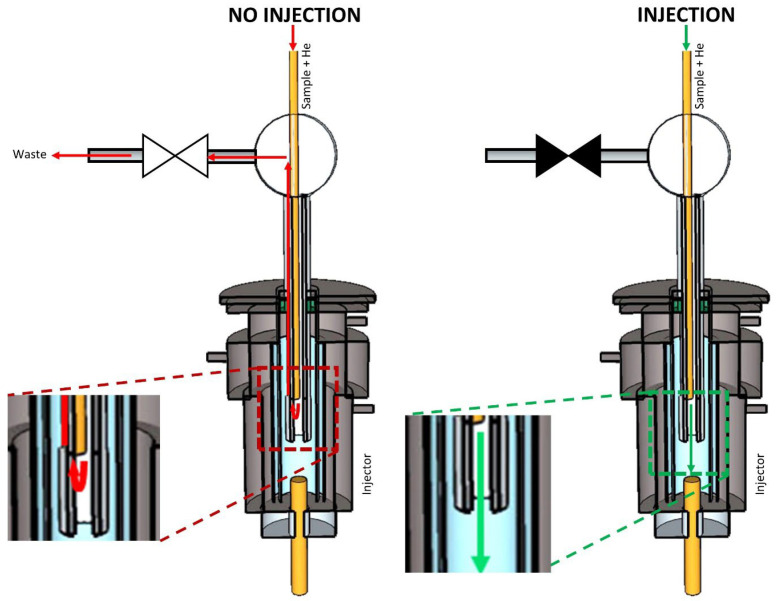
Scheme of multiplex injector design A. The green and red arrows indicate the flow of the carrier gas stream (transporting the sample) into and out of the GC’s split/splitless injector when the sample was introduced (‘Injection’) or not (‘No injection’). ▶◀ represents a solenoid valve in the ‘closed’ state, while ▷◁ represents the valve in the ‘open’ state.

For design A (Figure 2), a He makeup gas was added to the sample gas stream. Supported by this make-up gas stream, the sample was transported toward the GC through an uncoated capillary column. The capillary was fitted through a Tee connector and this also, into a hypodermic needle, which was inserted through the septum into the GC injection port. The capillary end was close to the needle’s end but still confined within the capillary. The other side of the Tee connector was connected to a normally open solenoid valve and through this to the waste. When the sample was not injected, the valve was opened, and the sample that eluted from the fused silica capillary was directed to waste because the GC column head pressure was higher than the pressure at the end of the transfer line, thus preventing the sample from reaching the GC column. In order to inject a portion of the gas stream, the valve was closed, which caused a back-pressure build-up. At one point, the backpressure created by the blocked flow path exceeded the column head pressure, and thus, the sample was injected into the GC column.

**Figure 3:**
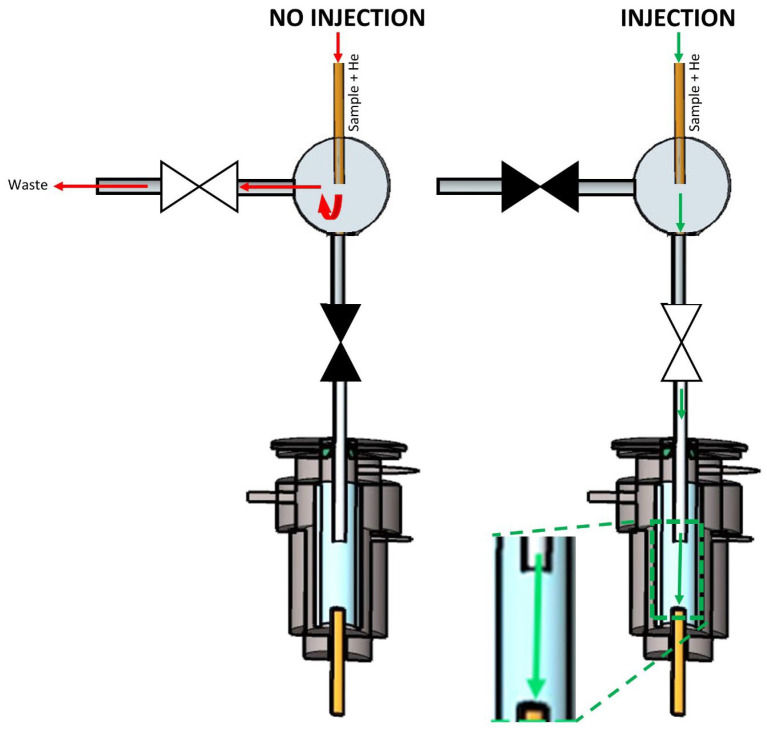
Scheme of multiplex injector design B. The green and red arrows indicate the flow of the carrier gas stream (transporting the sample) into and out of the GC’s split/splitless injector when the sample was introduced (‘Injection’) or not (‘No injection’). ▶◀ represents a solenoid valve in the ‘closed’ state, while ▷◁ represents the valve in the ‘open’ state.

Figure 3 shows the second design (design B), which is similar to the first, up to the point where the uncoated capillary reaches the Tee connector. The Tee connector was connected to a normally closed valve at the injection port side and a normally open valve at the waste side. When no injection was performed, the normally closed and normally open valves stayed in their default state. The sample that eluted from the capillary was directed to waste because the injection port side was closed. To inject, the normally open and closed valves were now closed and opened, respectively. This means that the flow path to waste was closed, and the sample was forced to the injection port side, which was then open.

**Figure 4:**
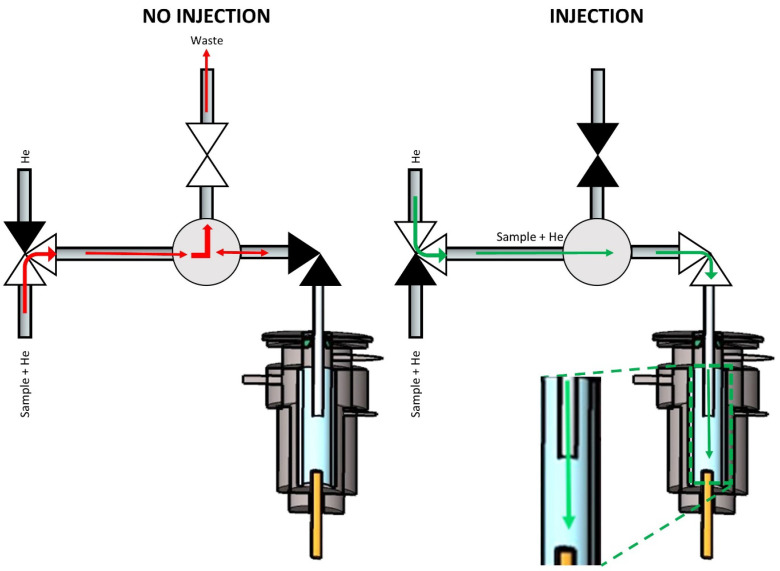
Scheme of multiplex injector design C. The green and red arrows indicate the flow of the carrier gas stream (transporting the sample) into and out of the GC’s split/splitless injector when the sample was introduced (‘Injection’) or not (‘No injection’). ▶◀ represents a solenoid valve in the ‘closed’ state, while ▷◁ represents the valve in the ‘open’ state. ▷^▼^◁ represents a three-way solenoid valve with the open symbols representing the open flow path and the closed symbol representing the closed flow path.

Design C (Figure 4) was different from the other two because it was a three-valve set-up that included one 3-way, one 2-way normally open, and one 2-way normally closed solenoid valve. The three-way valve was connected to the He supply from one side and to the sample, which was transported by He make-up gas from the other side. The third port was connected to a length of tubing that defined the sampling volume, similar to the sampling loop of a six-port valve. The tubing was connected via a Tee connector to the normally open valve leading to the waste and to the normally closed valve leading to the injection port. When not injecting, the sample flushed the tubing/loop while connected to the waste line. When injecting the sample, the 3-way valve switched from the sample to the He supply (kept at a higher flow than the sample). The normally open valve closed, and the normally closed valve opened. This led to the sample being injected into the GC column instead of going to waste since the flow path to waste was closed.

## 3. Results

The three different multiplexing injector designs described in the experimental section were mounted on a GC-BID instrument for further investigations. As a consequence of the multiplexed sample introduction, this system is capable of providing time-resolved chromatograms and improved time resolution. All three designs—denoted ‘Design A’, ‘Design B’ and ‘Design C’ in the following discussion—allow the computer-controlled introduction of a gaseous sample at precisely controlled intervals and sample introduction lengths.

At first, the investigation focused on whether each design was functional or not, and in the second stage, each system was optimized. The compound chosen for the initial testing was heptane, a compound in the middle of the boiling point range with respect to the intended application for LIB emission monitoring, which also shows very good peak shape and detectability with the BID detector. Different parameters and variations of the experimental setup were tested for their influence on the results. Among those were the use of a vacuum pump in the waste line, the inner diameter of the sample introduction capillary, the position of the sample introduction capillary inside the injector, the inner volume of the Tee connector and the length and inner diameter of the tubing connections.

### 3.1. Investigation of Experimental Setup

Initial experiments with different experimental setups were used with a vacuum pump in the waste flow line. The rationale behind this setup was to create a greater pressure drop between the column head pressure in the GC injector and the pressure at the end of the waste line and thus to efficiently prevent the leakage of sample from the sample introduction capillary into the GC injector during periods of no injection. For testing the usefulness of the pump, the conditions were a 50 °C oven temperature, 1 mL min^−1^ column flow, 20:1 split flow, 50 mL min^−1^ sample flow, 1 s injection time, tested pump flows: 0, 5; 15; 75; and 115 mL min^−1^ (measured at the outlet of the membrane pump). The experimental results showed that the use of the pump leads to irreproducible and somewhat variable amounts of sample being introduced into the GC (Supplementary Materials, Figure S3). The most probable reason for the peak area decreasing with an increasing pump flow rate is that the increasing suction created by the pump affects, i.e., reduces, the amount of sample introduced with each injection. Results were obtained successfully with short tubing connections with smaller inner volumes and without using a vacuum pump in the waste line. Short tubing lengths and small inner volumes were favorable as they resulted in faster sample introduction into the GC and more accurate results. In contrast, greater tubing lengths and larger inner volumes not only resulted in slower sample transfer but also gave rise to carryover between injections. While this problem can be resolved by increasing the sample flow or the injection time, this option is less attractive as it results in high gas and sample consumption. In the subsequent experiments, no pump was used in the waste line for either of the three injector designs. Maximum tubing lengths for designs A and B were 14 cm (inner volume: 90 μL) and 18 cm (inner volume: 100 μL), respectively. For design C, the tubing after the normally closed valve was as short as technically possible and with a small ID (inner volume: 55 μL) to avoid a memory effect from a not adequately flushed sample. The other tubing parts had larger IDs (inner volume: 130 or 530 μL). The three-way valve design mimics the function of a sampling-loop-based injection valve, in contrast to the other two designs, which perform time- and pressure-driven injections. The injected sample volume was calculated from the sample flow and injection time for the time-driven injections. When using lower sample flows, the smaller sample volumes did not disturb the equilibrium between the injector and the instrument, and the injection was achieved more easily. For a loop-based injection (and similarly, for design C, which mimics a loop-based injection), the loop volume defined the injected volume, which means that it had to be chosen adequately.

**Figure 5:**
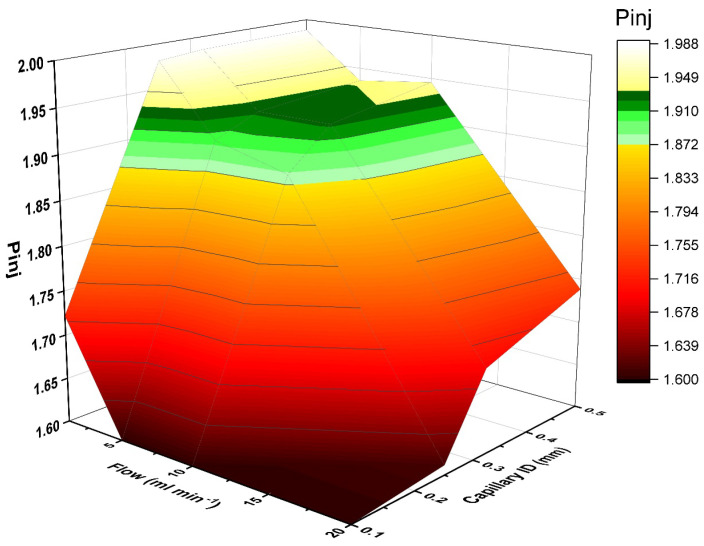
Interrelationship between sample flow rate, capillary ID and required injection pressure P_inj_ (in bar) for successful sample introduction.

Normally, in this system, the pressure that comes from the multiplex injector has to be higher or equal to the GC pressure (*P*_inj_ ≥ *P*_inst_). This is a prerequisite for a pressure-controlled sample introduction (see Supplementary Materials, Figure S4). With a multiplex injector, the pressure drop (Δ*P*) between the initial pressure before the sample (*P*_init_) and the pressure at the end of the injector (*P*_inj_) must be calculated. The pressure drop must not significantly affect the *P*_inj_. However, this was also determined by the requirements of the sample introduction. Figure 5 shows the relation between the *P*_inj_, the sample flow and the capillary ID. Values for *P*_inj_ were calculated for different combined flows of sample + He and capillary IDs, depending on the total tubing lengths and IDs according to the Hagen-Poiseuille equation. The parameter range, which is suitable for injection, was identified. The pressures were calculated at sample flows of 1-, 5-, 10- and 20-mL min^−1^ and 0.10-, 0.25-, 0.32- and 0.5-mm ID. As can be seen, there was a specific parameter range where injection could be successfully achieved (highlighted in color in Figure 5). This was always dependent on the column head pressure *P*_inst_ of the instrument. Thus, the accessible working range can change depending on the *P*_inst_.

**Figure 6:**
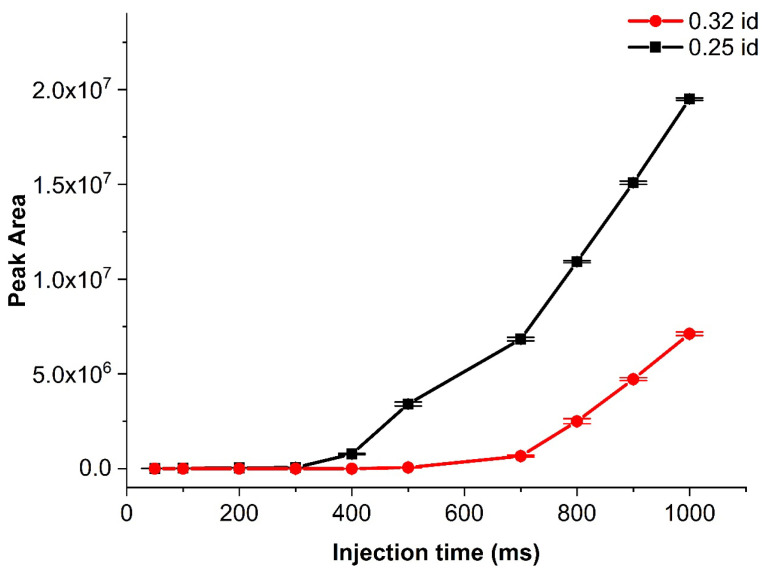
Comparison of the peak area of heptane for two sample introduction capillaries of different inner diameters (0.25 and 0.32 mm) with increasing injection time for design A. The isothermal method is mentioned in Section 3.3 with 60 mL min^−1^ sample flow. Intervals represent one standard deviation around the mean value.

To this end, the effect of capillary inner diameter (ID) for the first design was investigated. The investigation reported here was completed under isothermal conditions mentioned in Section 2.3. The tested capillary IDs were 0.10, 0.25 and 0.32 mm. Only the 0.25- and 0.32-mm IDs could be compared because, for the capillary with a 0.1-mm ID and the practically achievable pressure ratio, there was no or only a very small amount of sample injected. A possible reason for this behavior could be the high pressure drop between *P*_init_ and *P*_inj_, resulting in lower *P*_inj_ from the *P*_inst_. The comparison of the analyte signals from the corresponding chromatograms (Figure 6) showed that the 0.25-mm ID capillary produced much higher signal intensities for the same sample. Injections were achieved with injection times as short as 50 ms, but the corresponding sensitivity was low. Of note is that the injected amount increased only after 300 and 500 ms for the 0.25- and 0.32-mm ID capillaries, respectively. The trends looked similar. The only difference was the 0.32-mm ID capillary needed longer injection times to start reaching peak areas similar to those of the 0.25-mm ID column. The difference comes from the pressure drop, which resulted in different sample amounts being injected.

**Figure 7:**
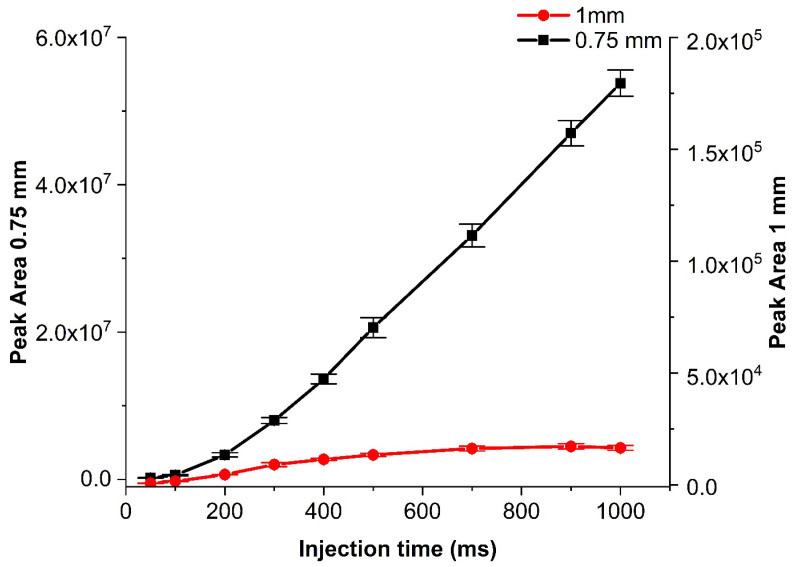
Comparison of the heptane peak area for two Tee connector bore sizes (1 and 0.75 mm) with increasing injection time for design B. The isothermal method is mentioned in Section 3.3 with a 7-mL min^−1^ sample flow. Error bars indicate ±1 s.

Another important aspect to investigate was the bore size of the Tee connector, which was important for all designs. Tested bore sizes (and corresponding dead volumes, in brackets) were 0.25 mm (0.47 μL), 0.75 mm (4.2 μL), 1 mm (7.5 μL), and 1.5 mm (34.8 μL). The bore sizes affected responses in two ways: With an increasing diameter, the dead volume of the connector increased, making the pressure build up slower and thus also injections slower, and even back-mixing possible. In turn, the pressure drop for the waste line was lower with an increasing bore size, allowing faster removal of the effluent gas stream, which in turn should make injection peaks narrower. Like with the capillary, we could not obtain results with the smallest bore size (0.25 mm), but we managed to compare the remaining ones and selected the 0.75 mm as the most appropriate for achieving higher intensity results for designs B and C (Figure 7) and all three bore sizes (0.75-, 1- and 1.5-mm ID) work successfully with different sample flow rates for the design A. The higher bore sizes worked well, but provided lower sample intensities under the same conditions. Therefore, an increase in the sample flow was needed for those. Unlike the capillary change, which is closely related to the pressure drop, and the structural changes in the bore size affect the flow, which then changes the injected sample amounts.

### 3.2. Investigation of Instrumental Parameters

**Figure 8:**
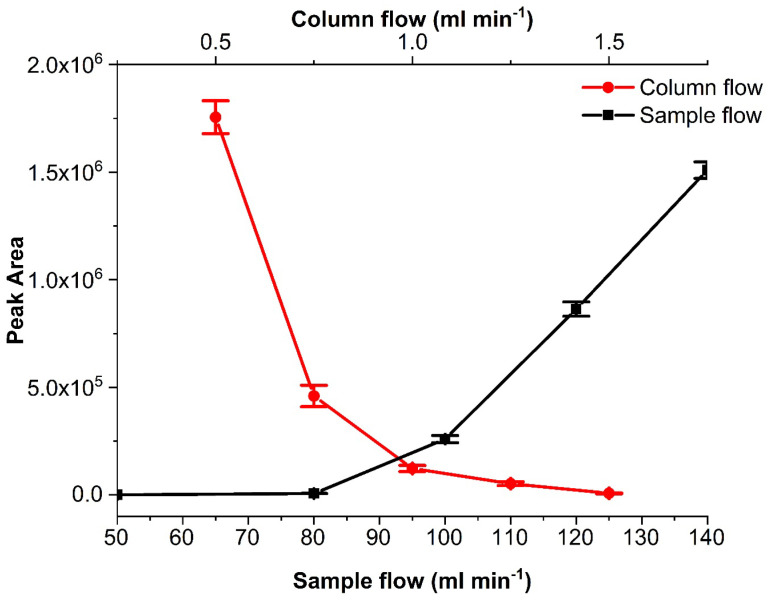
Heptane peak area changes at various columns and sample flows. The isothermal method is mentioned in Section 3.3. Error bars in the graph represent ±1 s.

Further instrumental parameters such as split ratio, column flow, sample flow, injection time, and time interval between injections were optimized. The tested split ratios were 10:1, 20:1, 75:1, 100:1, 200:1, 400:1 and 500:1, and column flows of 0.5, 0.75, 1, 1.25, 1.5 mL min^−1^. The higher the split ratio, the faster the sample was transferred to the GC column for a ‘normal’, i.e., liquid phase injection, where a large solvent vapor cloud was formed from the liquid volume injected. In the case of gaseous sample introduction for sub-second periods, this effect was not relevant, and the dilution effect of increasing split ratios was dominant. Hence, and also to reduce carrier gas consumption, a lower split ratio of 10:1 was adopted. Figure 8 demonstrates the effect of the column flow and sample flow on the peak area. Higher column flows moved the sample faster and, as can be seen, resulted in a lower response because of the change in the *P*_inst_ (Figure 8). The split ratio and the column flow were parameters that could change depending on the *P*_inst_ we wanted to achieve. In the present work, we needed to work with lower sample flows because our aim was to use the multiplex injector for the investigation of lithium-ion battery emissions, which (due to the small cell volume) allow only very low flow rates. So, the final split ratio was 10:1, and the column flow used was 1 mL min^−1^. An increase in the column flow or the split flow typically increases the column head pressure. An increased column head pressure will change the pressure situation between the front end of the sample introduction capillary and the GC injector. This may, in the extreme case, lead to a situation where there is no pressure drop toward the GC injector or, if the pressure drop is even inverted, result in no sample being injected. This demonstrates that the optimization of pressure and flow parameters in the multiplexing sample introduction device and the GC injector is delicate and decides whether the sample is successfully introduced or not into the GC. For the optimization of the further parameters, the conditions of GC flow and pressure were the same as reported before, with the only difference that during one run, a sample was injected multiple times (20 times). The investigated sample flows were 50, 80-, 100-, 120- and 140-mL min^−1^ with 50 ms injection time, with time intervals 0.5, 1, 2, 3, 4, 5, 6, 7, 8, 9 and 10 s between samples and the injection times were 10, 20, 40, 50, 100, 200, 300, 400, 500, 600, 700, 800, 900 and 1000 ms. For the first of our injector designs, lower sample flows (below 50 mL min^−1^) with the use of the 1.5 mm bore Tee union did not reach the necessary *P*_inj_. Higher sample flows showed faster sample introduction (due to the fact that the necessary pressure for injection was built up in a much shorter period of time), and thus, lower injection times of 50 ms could be used (Figure 8). With increasing sample flow, the pressure at the end of the sample introduction capillary increased above the column head pressure in the injector (*P*_inj_ > *P*_inst_), and more of the sample was introduced. If the *P*_inj_ is too high, this could result in sample overload. The flow must be high enough to ensure fast sample introduction at the ms time scale. The most appropriate time interval and injection time can be chosen depending on the selected flow. A short time interval (between injections) was desirable as it would determine the temporal resolution of the method. Longer interval times result in fewer data points of the measurement. However, very short interval times can result in significant peak overlap, which will be difficult to deconvolute. In our case, the lowest sampling interval time was 6 s (Table 1). In the case of the injection times, the higher they are, the higher the overload and broadened peak shape of the outcome. This was observed when using a high sample flow of 150 mL min^−1^. The peak asymmetry was 1.14 ± 0.03 at 50 ms and reached an asymmetry of less than 0.4 at injection times higher than 200 ms. The selected conditions for injector design A were 60–100 mL min^−1^ sample flow (depending on the bore size used), 6 s interval time, and 50 ms injection time. The injection time can vary from 50 ms to 1 s or more as it is a way to increase the injected volume. Similar tests were undertaken for injector designs B and C. With these, we managed to reduce the sample flow to 5–7 and 0.2–1 mL min^−1^, respectively. This decrease in sample flow rate became possible by the inclusion of an additional normally closed solenoid valve directly before the injector, which isolated the injector from the GC when the sample was not injected.

Further to the investigation of the used conditions, it was crucial to test whether the retention times were reproducible or not. Reproducible retention times are highly important for the deconvolution of the raw data with the Hadamard transform algorithm and other deconvolution algorithms developed for this work. This was tested after subsequently injecting the same amount of heptane 15 times into the system (three repetitive runs). In all cases, the interval between the injection times of two neighboring peaks was 0.01 min, with relative standard deviations less than 0.01%. For design A, the peak asymmetry was 1.09 ± 0.02, and the relative standard deviation (RSD%) for peak area and height was 3.8 and 2.9%, respectively. These favorable precision data also confirmed the injection time stability (in this case, 50 ms) since the amount of sample introduced was proportional to the injection time. Furthermore, experiments according to a 4-bit PRBS (15 injections) during different days showed RSD% values for the peak area and height of 8.2 and 8.4%. The peak asymmetry average values were 1.07 ± 0.01. For design B, the peaks for repeated injections of the same analyte constantly increased while the analyte’s concentration was constant. Even after taking measures against possible memory effects from the preceding injection, the relative standard deviation remained larger than 20%, and this design, therefore, was not used further for the experiments. For design C, the relative standard deviation (RSD%) for peak area and height was 3.3 and 6.2%, respectively. Experiments with random injections during different days showed RSD% values for the peak area and height of 8.4 and 4.2%. Peak asymmetry was found to be 1.28 ± 0.01. The advantage of design C over design A is that, while the latter, the system is easily affected by changes in the experimental set-up, the former offers the possibility of more accurate determination of the injected volume and, thus, more reproducible and accurate measurements. Additionally, with design C, the instrument was protected from unwanted sample injections during the start-up phase of the system, which was noticed with design A on another GC system.

### 3.3. Comparison to a 6-Port Switching Valve

Experiments were also performed with the 6-port gas sampling valve provided as a sample introduction option for the GC-BID instrument that was housed in a thermostatted valve box and compared to the designs we had already examined. The first drawback we faced was that all operations of the valve (injection, switching back) were limited by the time base of the instrument’s software. The smallest injection/switching time that could be set was 0.01 min (600 ms), which was much longer than the 50 ms achievable with the designs developed in-house. The second drawback consists of the relatively slow switching speed of the electrically actuated switching valve, which did not allow the use of loop filling/injection times of less than 0.02 min to inject the sample into the GC efficiently. It is evident that these problems could be resolved by controlling sample introduction externally, and by using a high-speed switching valve, but these options are not available at the present time. For the experiments that were performed with the switching valve, peak asymmetry was found to be 1.32 ± 0.017, and the RSD values for peak area and height were 0.8% and 1.2%, respectively.

### 3.4. Application to the Volatile Emissions from Lithium-Ion Batteries

**Figure 9:**
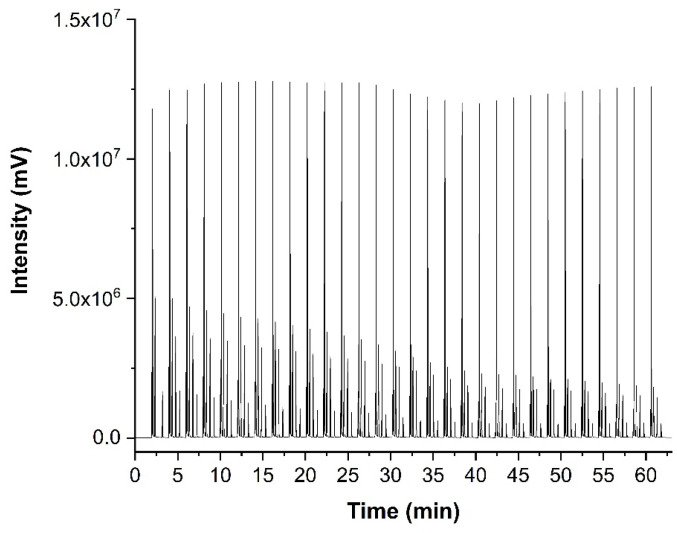
Multiplex chromatogram resulting from a dummy cell measurement with design C. The isothermal method is mentioned in Section 2.3. Seven compounds were analyzed.

**Figure 10:**
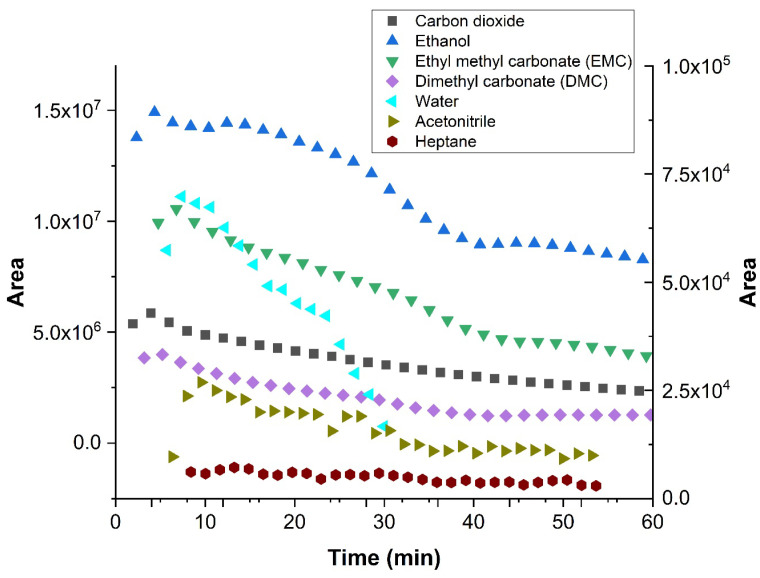
Change in concentration of dummy cell analytes during the experiment (30 injections in 60 min). The isothermal method, as specified in Section 2.3.

**Figure 11:**
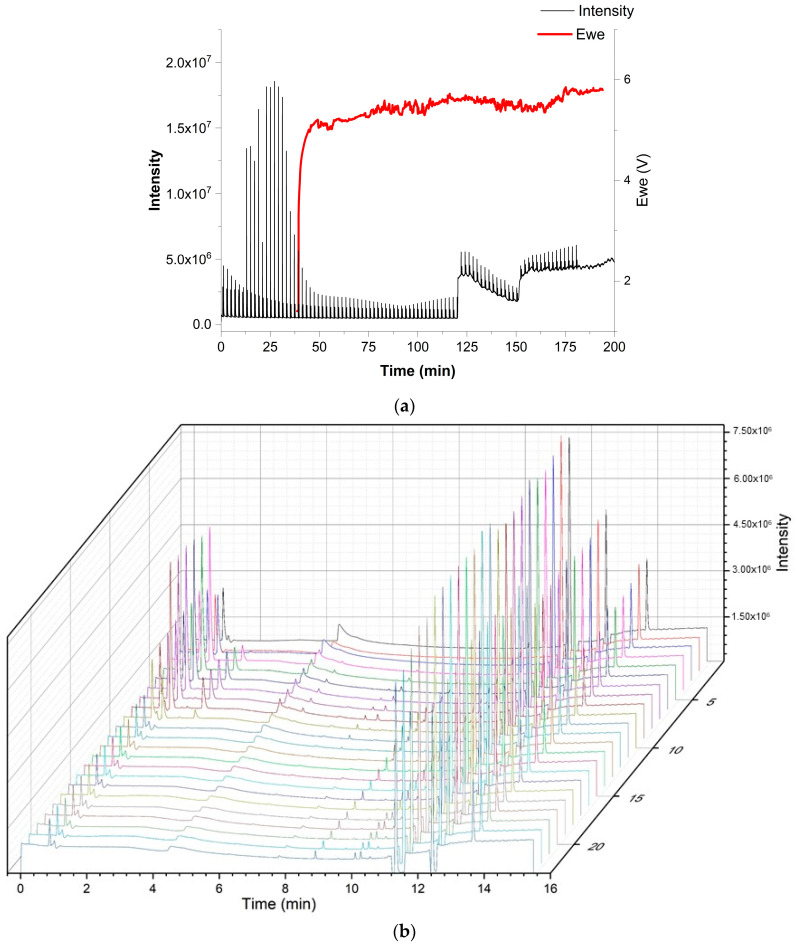
Real lithium-ion battery testing experiment of NMC 111 cell with the design C (**a**) (top) multiplexing and (**b**) (bottom) normal gradient conditions during overcharge. (E_we_: potential of a working electrode in V).

As a realistic test for the versatility of the above-described multiplexing sample introduction system for GC, the developed device was used to study the emissions of a dummy electrochemical cell with GC-BID, while measurements of the degradation products from the electrolyte of a lithium-ion battery by multiplexing-GC/MS were performed for safety reasons at another facility. A dummy cell containing the compounds of interest imitated the transient emission of typical battery electrolyte degradation products. It initially included five compounds: ethanol; ethyl methyl carbonate (EMC); dimethyl carbonate (DMC); acetonitrile; and heptane. Air (O_2_ + N_2_), CO_2_ and water leaked into the dummy cell at a later stage or were created from the electrolyte decomposition. The tests were performed with injector design C. Sample injections were performed every 2.02 min in order to obtain a multiplex chromatogram (Figure 9). These were subsequently processed and produced chromatograms, which showed the peaks attributable to seven individual compounds. Figure 10 depicts the peak area change in each compound over time and imitates the battery discharging state where most degradation products show a decrease. The test demonstrated that the multiplex injector and the data processing can successfully handle even complex samples. The investigation was continued by coupling the multiplex injector to a GC-MS instrument for the overcharge experiments (conditions specified in Section 3.3). The tested cell was assembled from am NMC 1:1:1 (Li_1–x_(Ni_0.33_Mn_0.33_Co_0.33_)O_2_) lithium nickel-manganese-cobalt (1:1:1) oxide cathode prepared vs. a graphite anode in an EL-Cell. During the overcharging experiment, the battery voltage was increased by applying constant current (CC) above its recommended maximum potential. The testing was undertaken after performing a specific constant current–constant voltage (CCCV) formation cycle. This experiment—in which the particular sample introduction device that was previously mounted on the GC-BID system was installed on a GC-MS system—demonstrated that the multiplex injector unit is easily transferable to other instruments and allows comparing the results with the standard sampling method that is usually used. Overcharging experiments were conducted until 250% of the rated capacity. This was achieved by applying the 1C rate for fast overcharging while using multiplex sampling (180 chromatograms were recorded in three hours). In contrast, the monitoring of degradation products by temperature-programmed separation was significantly slower, where the overcharging was produced by a C/3 rate (24 chromatograms in 12 h, Figure 11b). For the 3 h experiments, a drift in the baseline was observed without having a significant effect on the peak identification (Figure 11a). The change is attributed to the high amount of solvents emitted from a Li-ion battery, which continuously accumulate as samples and are constantly introduced into the system. From real battery testing, useful information on the battery degradation products was obtained during overcharging. The compounds detected and investigated for their concentration change over time were CO_2_, ethane, water, acetaldehyde, fluoroethane, DMC and methyl formate. The overcharge process resulted in an increase in CO_2_, fluoromethane and methyl formate formation.

## 4. Conclusions

In the current study, we developed three different designs for multiple sample introduction into a GC system. The operational characterization of the three devices showed that two of the designs (A and C) successfully worked after optimization of the respective working conditions. This included the parameters sample flow, column flow and injection time on which the analytical result critically depends, particularly. Another important aspect of the success of the proposed setup was the solenoid valve which had to be fast-switching and had a very small dead volume.

The multiplex system developed in this study provides the advantage of repeated sample introduction in a s short time, which, together with the in-house developed software for data deconvolution, allows the acquisition of a significantly larger number of chromatograms per given time and thus obtains a larger amount of information for a transient process compared to sequential data acquisition. The multiplexing injector is self-confined and can be installed on different instruments, for example, GC with atmospheric pressure detectors such as the BID or even with vacuum detection such as a mass spectrometer (adjustment of the optimum operating parameters is required then).

Finally, GC with multiplex sample introduction was applied in the monitoring of LIB degradation products. Recording the chromatograms in shorter intervals along the transient process allowed for a better understanding of the reactions that take place under normal or abusive conditions and identifying hazardous compounds and their concentration changes.

## Figures and Tables

**Table 1:** Analytical characteristics of design A for different interval times (Δt).

Interval Time [s]	Peak Area ^1^		Peak Height ^1^		Asymmetry ^2^	
	Mean	RSD%	Mean	RSD%	Mean	RSD%
2	693,516	5.6	338,558	6.7	-	-
3	700,262	2.2	297,074	0.5	-	-
4	698,850	0.9	287,342	0.8	-	-
5	694,628	0.8	286,460	1.2	0.75	1.5
6	672,199	3.0	281,610	1.3	1.13	2.8
7	667,479	1.0	281,128	0.8	1.14	1.5
8	680,908	0.5	285,048	0.4	1.16	0.0
9	681,597	1.0	283,220	1.1	1.13	3.2
10	797,033	2.0	330,935	2.7	1.11	3.0

^1^ Mean and RSD% values were calculated from 5 data points. ^1^ Where asymmetry values are not reported, peaks are not resolved at 10% of the peak height and asymmetry can consequently not be calculated.

## Data Availability

The raw data supporting the conclusions of this article can be made available by the authors on request.

## Supplementary Materials

The following supporting information can be downloaded at: https://www.mdpi.com/article/10.3390/moleculesXXXXX/s1, Figure S1: Graphical representation of the production of a multiplexed chromatogram; Figure S2: Algorithm for the deconvolution of multiplexed chromatograms; Figure S3: Change of peak area response with increase of pump flow in the waste line; Figure S4: Sketch of the different pressures within the GC system in order to achieve a pressure-driven injection into the GC; Figure S5: Construction drawing (left and middle) and photo (right) of the actual set-up of the multiplex injector (Design C), mounted on the rear injection port of a Shimadzu 2010 GC.
