# Supplementary material for: Development of a Multiplexing Injector for Gas Chromatography for the Time-Resolved Analysis of Volatile Emissions from Lithium-Ion Batteries
Source: Molecules. 2024 May 7;29(10):2181. doi: 10.3390/molecules29102181 (PMC11123839; doi:10.3390/molecules29102181)
Supplement: Supplementary file 1 [file molecules-29-02181-s001.zip › molecules-2834078-supplementary.pdf]

## Supplementary Information for the manuscript

# Development of a multiplexing injector for gas chromatography for the time-resolved analysis of volatile emissions from lithium-ion batteries

**Maria Antoniadou [1], Valentin Schierer [2], Daniela Fontana [3], Jürgen Kahr [2], Erwin Rosenberg [1*]**

1 Vienna University of Technology, Institute of Chemical Technologies and Analytics, Getreidemarkt 9/164, A-1060 Vienna, Austria
2 Austrian Institute of Technology GmbH, Electric Drive Technologies, Electromobility Department, Giefinggasse 2, A-1210 Vienna, Austria; valentin.schierer@tuwien.ac.at (V.S.); juergen.kahr@ait.ac.at (J.K.)
3 FAAM Research Centre, Strada del Portone 61, I-10137 Torino, Italy

* Correspondence: erwin.rosenberg@tuwien.ac.at (E.R.)

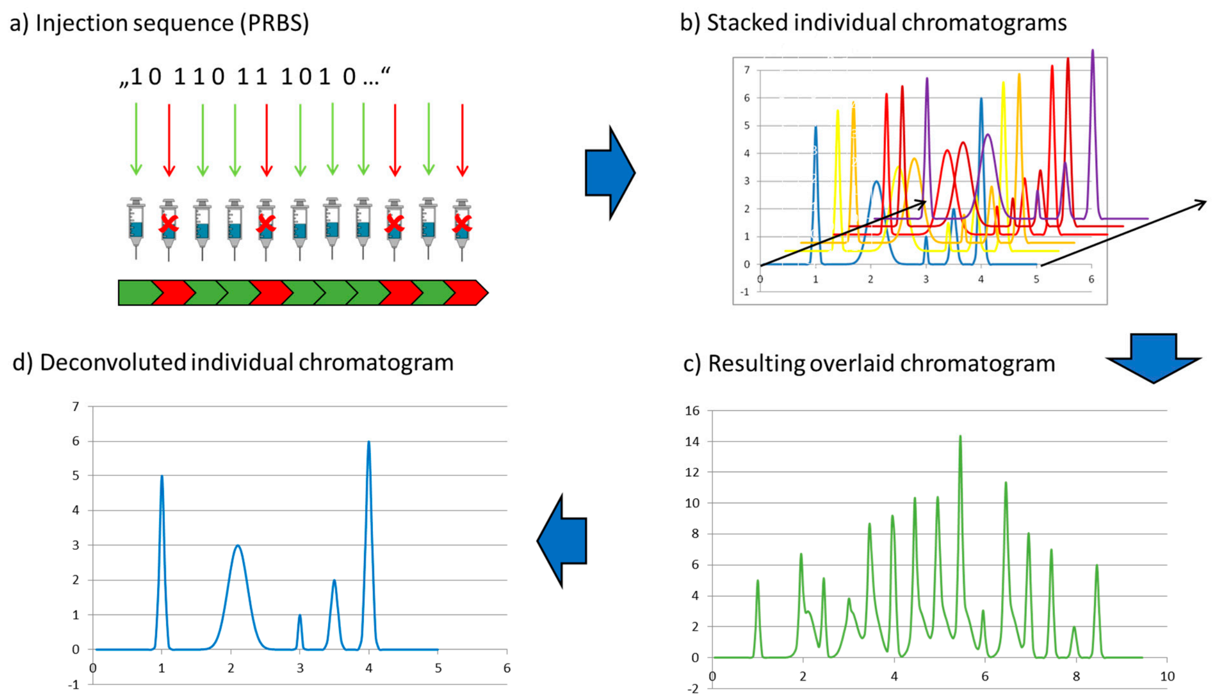


**Figure S1.** Graphical representation of the production of a multiplexed chromatogram: *a)* sample is injected at different, pred-defined times in a pseudo-random binary sequence (PRBS) for later deconvolution by the Hadamard (back-)transform, or in regular intervals if deconvolution by subtraction is applied; *b)* the individual chromatograms overlap to form *c)* a multiplexed chromatogram. This chromatogram can be deconvoluted to *d)* re-calculate the individual original chromatograms.

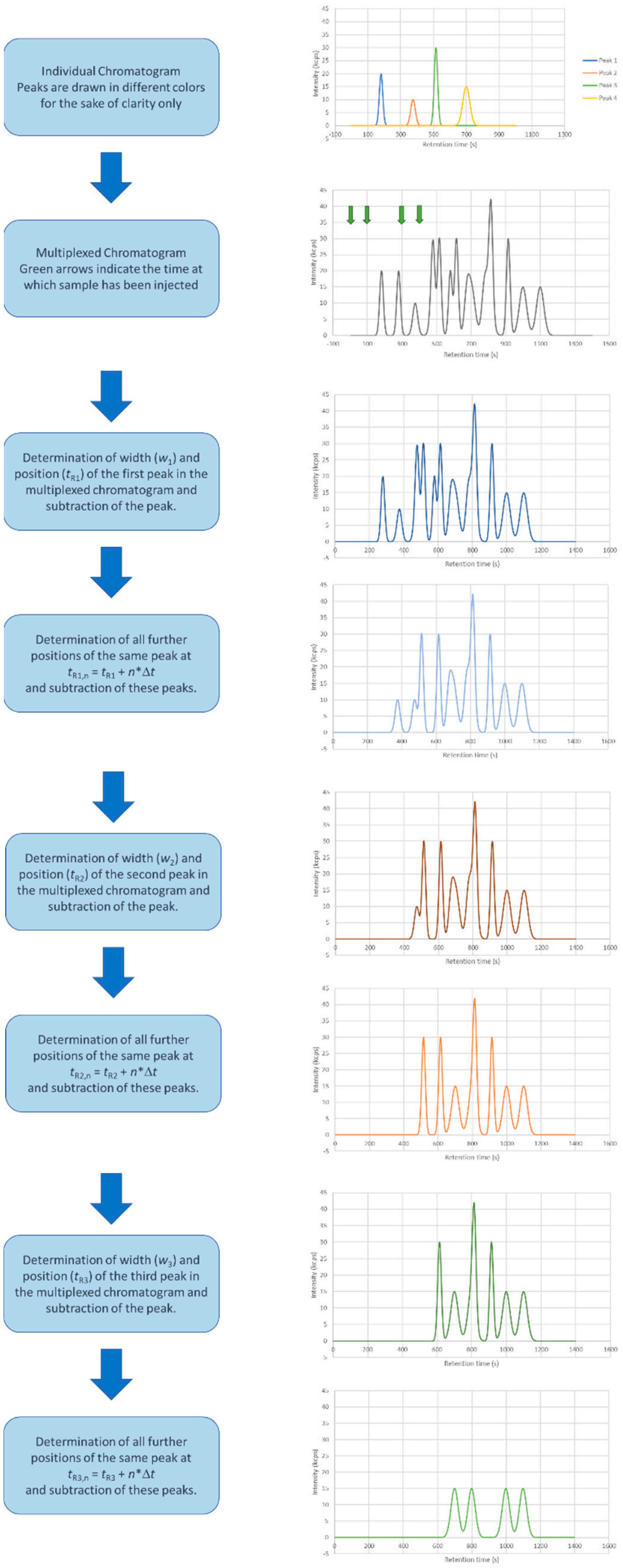


**Figure S2.** Algorithm for the deconvolution of multiplexed chromatograms, based on *i)* determination of peak width ($w_i$) and retention time ($t_{R1}$) of the first peak and *ii)* iterative subtraction of all appearances of this peak. Next, the second, third and all subsequent peaks are subtracted, based on the knowledge of the injection sequence (green arrows indicate injection). The fit of the intensity of the subtracted peaks ($k_i$) is the concentration relative to the reference chromatogram.

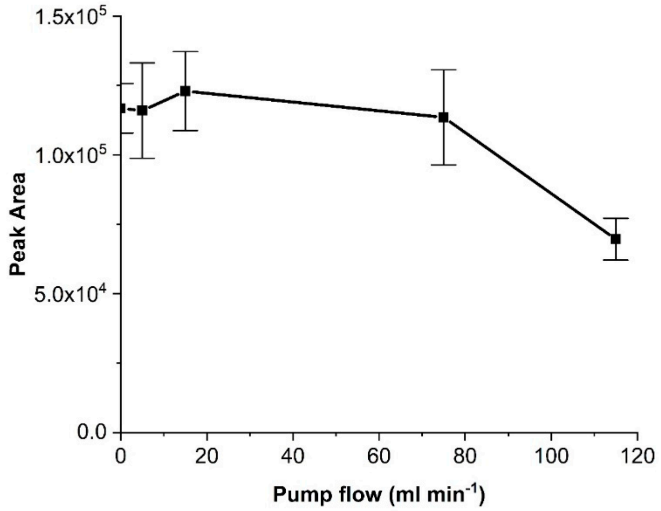


**Figure S3.** Change of peak area response with increase of pump flow in the waste line. (Design A, constant column head pressure and isothermal program mentioned in section 3.3. of the manuscript) Error bars in the graph indicate ±1 standard deviation.

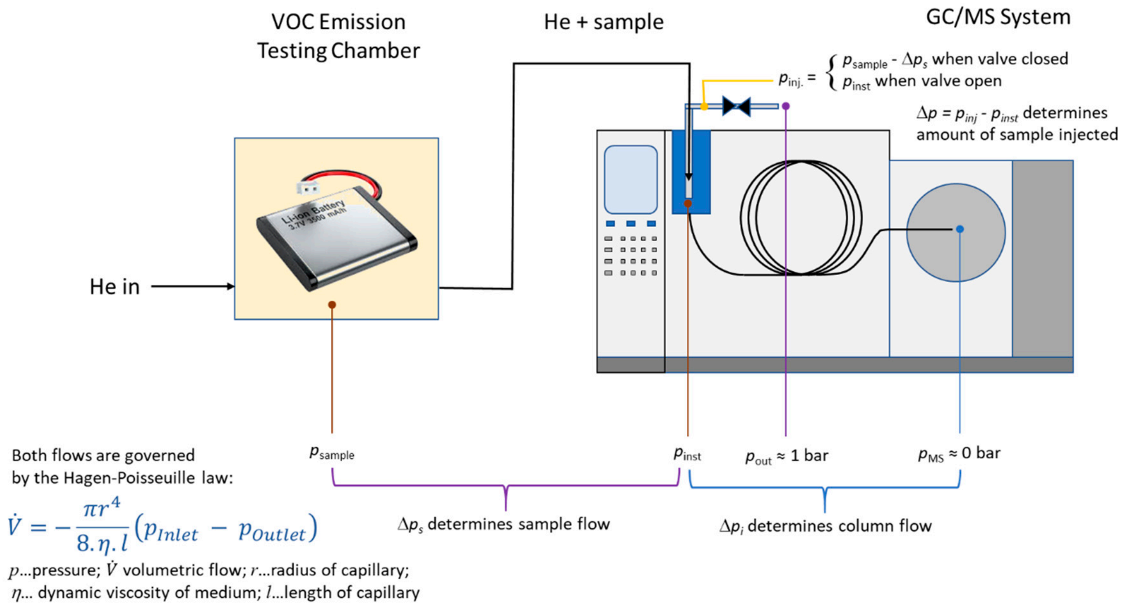


**Figure S4.** Sketch of the different pressures within the GC system in order to achieve a pressure-driven injection into the GC.

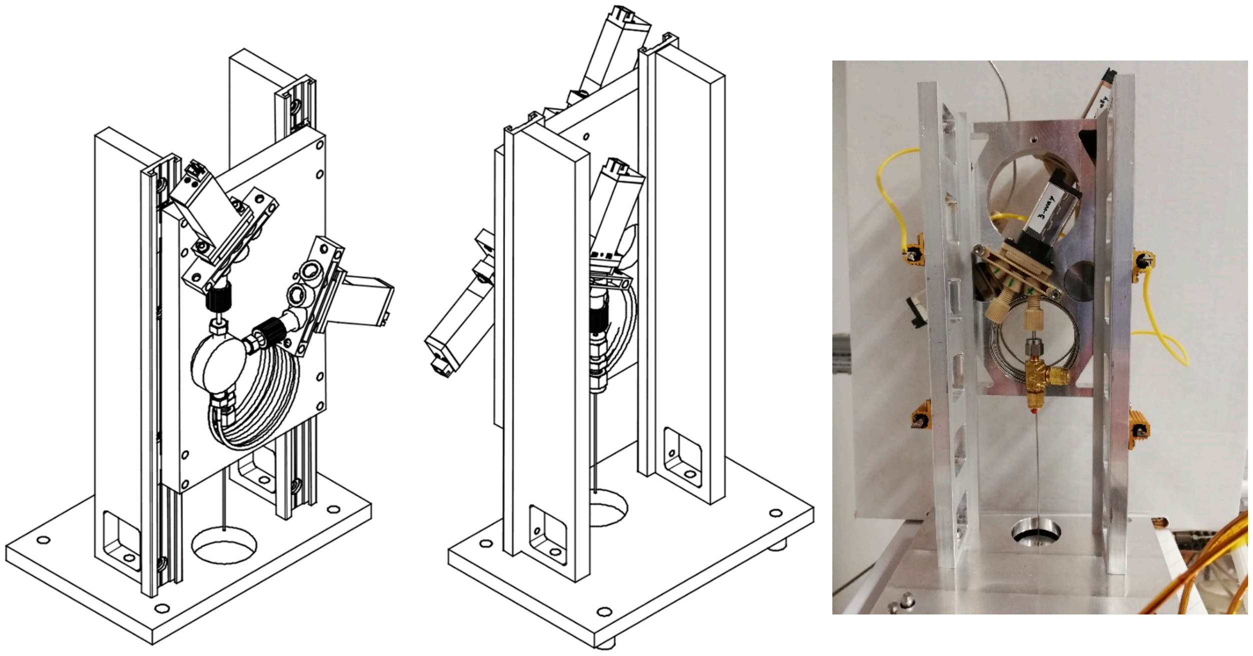

**Figure S5.** Construction drawing *(left and middle)* and photo *(right)* of the actual set-up of the multiplex injector (Design C), mounted on the rear injection port of a Shimadzu 2010 GC. (Drawings provided by J. Frank, Central Workshop of the Faculty of Technical Chemistry, TU Wien.)
